# Vigorous Dynamics Underlie a Stable Population of the Endangered Snow Leopard *Panthera uncia* in Tost Mountains, South Gobi, Mongolia

**DOI:** 10.1371/journal.pone.0101319

**Published:** 2014-07-09

**Authors:** Koustubh Sharma, Rana Bayrakcismith, Lkhagvasumberel Tumursukh, Orjan Johansson, Purevsuren Sevger, Tom McCarthy, Charudutt Mishra

**Affiliations:** 1 Snow Leopard Trust, Seattle, Washington, United States of America; 2 Nature Conservation Foundation, Mysore, Karnataka, India; 3 Panthera, New York, New York, United States of America; 4 Snow Leopard Conservation Foundation, Ulaan Baatar, Mongolia; 5 Grimso Wildlife Research Station, Swedish University of Agricultural Sciences, Uppsala, Sweden; University of Florence, Italy

## Abstract

Population monitoring programmes and estimation of vital rates are key to understanding the mechanisms of population growth, decline or stability, and are important for effective conservation action. We report, for the first time, the population trends and vital rates of the endangered snow leopard based on camera trapping over four years in the Tost Mountains, South Gobi, Mongolia. We used robust design multi-season mark-recapture analysis to estimate the trends in abundance, sex ratio, survival probability and the probability of temporary emigration and immigration for adult and young snow leopards. The snow leopard population remained constant over most of the study period, with no apparent growth (λ = 1.08+−0.25). Comparison of model results with the “known population” of radio-collared snow leopards suggested high accuracy in our estimates. Although seemingly stable, vigorous underlying dynamics were evident in this population, with the adult sex ratio shifting from being male-biased to female-biased (1.67 to 0.38 males per female) during the study. Adult survival probability was 0.82 (SE+−0.08) and that of young was 0.83 (SE+−0.15) and 0.77 (SE +−0.2) respectively, before and after the age of 2 years. Young snow leopards showed a high probability of temporary emigration and immigration (0.6, SE +−0.19 and 0.68, SE +−0.32 before and after the age of 2 years) though not the adults (0.02 SE+−0.07). While the current female-bias in the population and the number of cubs born each year seemingly render the study population safe, the vigorous dynamics suggests that the situation can change quickly. The reduction in the proportion of male snow leopards may be indicative of continuing anthropogenic pressures. Our work reiterates the importance of monitoring both the abundance and population dynamics of species for effective conservation.

## Introduction

Population monitoring programs form an important component of conservation efforts. They provide information on the status of wildlife populations and can help evaluate the effectiveness of conservation actions, thereby allowing for adaptive management. In addition to population trends, population monitoring programs can help better understand the biology of the species, especially the fundamental processes of survival, reproduction and temporary emigration and immigration, along with their associated vital rates [1]. Empirical estimates of various vital rates such as survival, recruitment, and migration can provide insights into population performance, connectivity, and vulnerability to external threats [2], [3].

Large carnivores play an important functional role in ecosystems, are often the focus of conservation programs, and yet, monitoring their population dynamics has remained challenging. Their large home ranges tend to make them useful umbrella species, whose conservation can help protect several other species and large habitats [4]. Additionally, the charismatic image of large carnivores often incites strong emotions, making them suitable symbols for management of large ecosystems. With a growing human population and its ecological footprint, anthropogenic activities come into direct conflict with the ecological needs of large wide-ranging carnivores [5]. Low densities [6], large home ranges [7], and elusive behaviour make it difficult to monitor large carnivore populations in their natural environment. Non-invasive methods, such as camera trapping [8] and fecal genetics [9] can provide statistically robust population estimates of large carnivores. For felid species with individually distinct fur patterns, such as tigers *Panthera tigris* [8], jaguars *Panthera onca* [10]–[12], snow leopards *Panthera uncia* [13], leopards *Panthera pardus* [14], cheetahs *Acinonyx jubatus* [15] and ocelots *Leopardus pardalis* [16], [17], data from camera-trapping can be analysed using capture-recapture models to estimate abundances and population dynamics [2].

The snow leopard is a globally threatened large carnivore distributed over an area of c. 2 million km^2^ in the mountain ecosystems of 12 countries in South and Central Asia. It is believed that their global population may be between 4,000 and 6,500 in the wild, though there is no robust basis for these estimates [18]. The species is classified as endangered by the IUCN. Retaliatory persecution by pastoralists, depletion of wild prey, habitat degradation, poorly planned developmental activities, extractive industries such as mining, and poaching pose the main threats to the species' survival. The snow leopard enjoys the highest legal protection in the 12 range countries where it is found and considerable conservation efforts are being made towards improving their status.

A few studies have been conducted on snow leopards to estimate their population using mark-recapture framework on camera trapping data (e.g. [13], [19], [20]), but these have been single-season efforts, and robust design population analyses based on time-series data are not available for any snow leopard population (but see [13]). Based on four years of camera trapping in the Tost Mountains of South Gobi, Mongolia, we describe, for the first time, the abundance and dynamics of a wild snow leopard population over multiple years. We examine the trends in population abundance and sex ratio, and estimate the detectability, survival probability and probabilities of temporary emigration and immigration for adult and young snow leopards. Our results bring to light vigorous underlying dynamics in an otherwise ‘stable’ population of an endangered large carnivore.

## Study Area

Tost mountain range is situated in the Tost *Bag* (smallest administrative unit in Mongolia) of the South Gobi province in Southern Mongolia (43.2°N, 100.5°E) and comprises of Tost and Tosonbumba Mountains. Temperatures here range between −35°C in the winter and +35°C in the summer. Water is a limiting resource during summer as most waterholes dry up leaving only a few perennial springs to sustain wildlife. Some man-made wells support livestock and people, though even they often rely on springs especially through summer. The altitude ranges between 1600 m and 2400 m above mean sea level and although not very high, the mountain slopes are characterized by steep cliffs and crevices. The study area is surrounded by steppe on three sides with a few isolated rugged hillocks potentially creating narrow corridors to habitats in the north and west. The Nemegt Mountains, part of the Gurvan Saikhan National Park, are separated by 60 km of steppe north of the study area. Towards the west, the study area is connected to the Great Gobi ‘A’ Strictly Protected Area through a broken network of small hillocks in the Trans Altai Gobi desert, with the largest gaps in between the hillocks being c. 25 km. The nearest mountain range in the south is separated by the international border with China, delineated by a trench and an approximately 400 km wide steppe of the Alashan Gobi desert. Towards the east, there is prominent human presence in the form of roads, mining establishments, and the Gurvantes township, followed by the Noyon Mountains.

**Figure 1:**
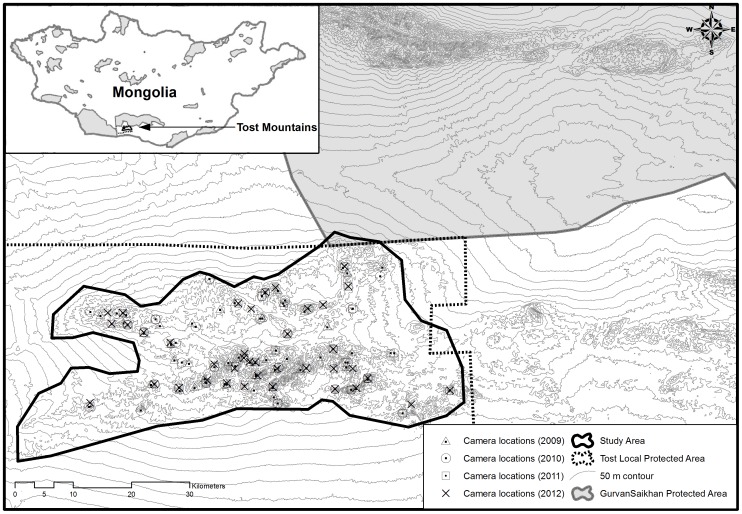
Location of camera traps from 2009 to 2012 in the Tost Mountains in South Gobi, Mongolia, where the population monitoring study of snow leopards was conducted. The camera locations were adjusted every year within the study area after conducting fresh surveys to ensure best camera placements for the year.

Legally, the entire study area now lies within the 7,000 km^2^ Tost Local Protected Area that was declared in 2011 (Fig. 1). The human population of Tost Bag was 935, according to a census in 2012. Goats and sheep are the most abundant livestock with 31,400 heads, followed by camel (1,100 heads), horse (120 heads), and cattle (47 heads). The Snow Leopard Trust and Snow Leopard Conservation Foundation have been implementing community-based conservation programs in the study area for several years. Ibex *Captra sibirica* is the primary prey of snow leopards in this region, followed by domestic goat *Capra hircus*, argali *Ovis ammon*, domestic sheep *Ovis aries* and other species including Tolai hare *Lepus tolai*, Chukar partridge *Alectoris chukar*, and several species of rodents ([21], Johansson, unpublished data). Other predators include lynx *Lynx lynx*, wolf *Canis lupus*, red fox *Vulpes vulpes* and at least two species of mustelids.

## Methods

### Field

We defined our study area as the entire mountainous region of Tost that was separated from other mountains by at least 20 km of steppe (Fig. 1). This comprised a surface area of 1,684 km^2^. We sought all necessary permits from the Mongolian Ministry of Environment and Green Development to conduct research in the Tost Mountains. The methods for the study were approved by the Mongolian Institute of Biology and the work detailed here did not involve any direct sampling methods or the collection of any specimens.

**Figure 2:**
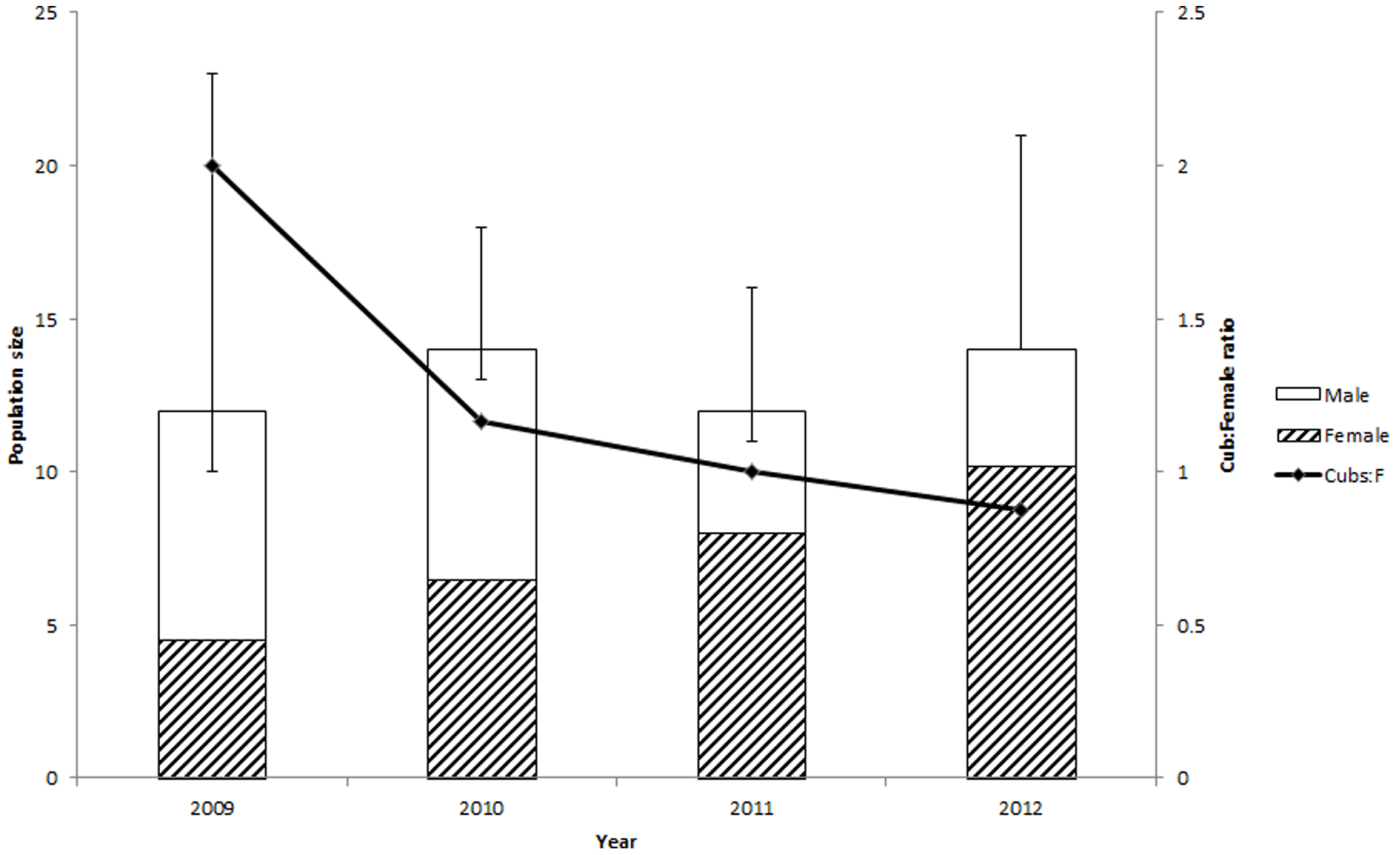
Trends in sex ratio and size of the adult population of snow leopards in the Tost Mountains, South Gobi, Mongolia, between 2009 and 2012. Population size was estimated independently over 4 years using the Jackknife Heterogeneity Model (Mh). Also shown on the secondary axis is the ratio of cubs per adult female. It is worth noting that the number of cubs recorded remained constant even though the ratio declined.

Data from the on-going study in Tost involving 25,297 locations from 19 radio-collared snow leopards indicated that snow leopards did not use the steppe, except for occasional forays or to migrate to neighbouring mountain ranges. We overlaid a grid of 5×5 km on the mountainous part of the study area to aid placement of one camera trap station in each grid cell. This size was deemed appropriate allowing about 2 camera stations per home range [22] since the mean minimum summer and annual ranges of snow leopards in Tost are estimated to be c. 55 km^2^ and 70 km^2^, respectively for 9 adult breeding females using fixed Kernel estimators on 12,498 locations over a period of five years (Johansson et. al. *unpublished data*). In each grid cell we searched along saddles on the ridgelines, valley or cliff bases and near overhanging boulders for fresh signs of snow leopard presence such as urine spray markings and scrapes. Within each cell, the site with most signs of recent activity was chosen and a camera trap was installed. Camera placements were rarefied if certain grid cells did not have ample suitable habitat or signs of snow leopard presence. The resultant density varied between 2 and 4 camera trapping stations/100 km^2^. Overall, 40–42 camera trapping stations were operated each year with one camera trap (Reconyx RM45, RC40 or HC500) placed at each (Fig. 2). Camera traps were left in the field for at least 62 days each summer, except in 2009 when due to logistic constraints, cameras were operational for 32 days. Camera traps were checked once every two weeks to ensure battery and memory card adequacy. We used at least 2 Gigabyte cards, allowing each camera to store more than 10,000 pictures. In total we invested a sampling effort of 8,490 camera-trap-nights over 4 years.

**Figure 3:**
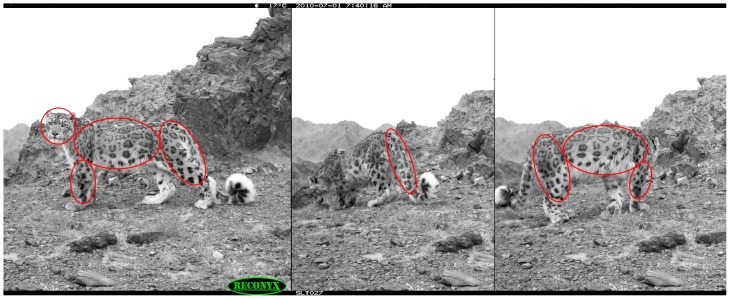
Multiple images with more than one body part of the snow leopard could be photographed using a single camera trap capable of taking multiple images rapidly (<1 second intervals). Here we show 3 out of the 30 different photos of a male snow leopard taken on the same encounter during the 2

We installed camera traps in sites where snow leopards were expected to spend more time (for marking or investigating) than just passing through. We programmed the camera traps to take 5 pictures on each trigger with an interval of about 0.5 seconds between each picture. The time period between two triggers was kept as zero. This meant that if the activity that triggered the first set of pictures continued after the first five pictures were taken, then another set of five pictures was taken immediately. This allowed us to get multiple pictures (range 1 to 115) of individual snow leopards for most captures (99%) and on many occasions we were able to photograph combinations of face, tail, foreleg, hind leg, and/or flank during the same encounter (Fig. 3). The trigger sensor on Reconyx cameras are calibrated to take pictures using a combination of heat and motion sensors that prevent unnecessary triggers due to heated rocks or movements of grass blades in front of the camera's field of view.

### Analysis

#### Snow leopard identification

Images were downloaded at the end of each sampling period. Markings on the face, rump, tail and flanks were used to identify individuals using pictures obtained during camera trapping.

Snow leopards have thick fur that tends to get ruffled up, which can lead to misidentification. Misidentification of an individual as two different individuals can result in an underestimation of detection probability and increase the minimum population count (number of individuals captured). Together, this can lead to an exponential overestimation of the population. To prevent this, we did not consider any two snow leopard images as belonging to different individuals unless at least three marking patterns were confirmed to be different. Conversely, images with three or more similarities were considered to belong to the same individual. In cases where we failed to find at least three markings that were either similar or different, the images and the particular encounter were discarded from the analyses. Images from different years were also compared with each other to identify individuals. Many images that remained unidentified in the initial years could be identified by the end of the fourth year.

#### Abundance estimation

Single season mark-recapture analysis allows for sampling designs where a study area is sampled in parts and analysed as a whole (e.g. [23], [24]). Although we sampled the entire area during the same period, this flexibility allowed us to use the entire data from a camera trap right from the day of installation, irrespective of whether or not the other camera traps had become operational. We used single season models to estimate the adult population each year using a 32 days' dataset in 2009 and up to 62 days' datasets for the remaining years using software CAPTURE [25]. We tested for possible effects of the shorter sampling duration in 2009 by randomly truncating the datasets from the subsequent years to 32 days. We found that the shorter sampling duration increased the variance of the estimates but did not influence the means, and therefore, used the longer data-sets starting 2010.

We created capture histories in the standard X-matrix format [25] by pooling the one-day sampling occasion capture histories into two-day sampling occasions capture histories. Population closure was tested using program CAPTURE [25]. The snow leopard population size was estimated independently for each year. To assess the accuracy of our population estimates, we separately analysed the photographic capture data of radio-collared snow leopards in the same study site, and compared our mark-recapture estimates for radio-collared animals each year with the known number of individuals wearing collars during the respective sampling period.

We tested whether the population had remained constant over the four primary sampling periods using robust design as well as Pradel's models [26]. The robust design model enabled us to test the hypothesis that the population remained constant, while the Pradel's model allowed us to assess the rate of change in abundance (λ) over the four annual intervals (2009–2012).

#### Estimating life history parameters

Robust design multi-season mark-recapture modelling [27] was used to estimate life history parameters such as survival, temporary emigration and immigration, and changes in abundance. Since camera setup in the field took up to 15 days, and about a week for retrieval, we truncated the data to the days when all cameras were operational for the multi-season analysis. This resulted in a secondary sampling period of 16 days for 2009, and 32 days each for 2010, 2011 and 2012. For each primary sampling period (year) we thus obtained 8, 16, 16 and 16 secondary sampling periods, respectively, by pooling the one-day sampling occasion capture histories into two-day sampling occasions capture histories. The snow leopard populations were expected to be open to changes between primary sampling periods (i.e. years) while we assumed the population to be closed to changes between secondary sampling periods.

Survival probability was estimated as the probability that an individual survived between two primary sampling periods. Modelling of capture-recapture data from multiple seasons using robust design allows estimation of probability of temporary emigration and immigration [27], [28]. This addresses the issue of individuals that survived and were unavailable for camera trapping during the subsequent primary sampling period(s) (considered temporarily emigrated from the study area), but were available again at a later primary sampling period (considered immigrated back into the study area). We tested the probabilities of temporary emigration and immigration to be random as well as Markovian. Markovian models expect that the probability of an individual being in a particular state (within the population or as a migrant) is dependent on its previous state, whereas random models expect that the probabilities of temporary emigration and immigration are independent of the previous state [28]. Detection probability was estimated for individuals present in an area but not encountered (captured on cameras) during the secondary sampling periods. Unlike the proportion of known individuals that were assumed to have temporarily emigrated and unavailable for capture, this addressed the proportion of individuals that were encountered (or not encountered), given presence.

We grouped encounter histories into two categories for multi-season analysis of survival and temporary emigration and immigration - individuals first time marked as young (cubs accompanying mother) and first time marked as adult. For individuals marked as young, the survival and emigration-immigration probabilities were modelled to change after two years, by which time they were expected to have grown into independent adults (or sub-adults). Although it is relatively difficult to sex snow leopards from their pictures given their thick fur, over the years of sampling, we were able to correctly sex all adult snow leopards based on visible testicles, presence of young cubs, and through information gathered during snow leopard captures for our telemetry study. Model based inferences from a separate analysis using adult snow leopards' data, grouped as male and female on program Mark indicated that the detection probability did not vary between sexes. We therefore used this information for estimating annual adult sex ratios, but did not use it to estimate additional population parameters due to further fragmentation of the small sample size.

Using robust design analysis of multi-season data, we developed several models to understand the effects of different variables on the modelled parameters that included survival probability, temporary emigration and immigration, and capture and recapture probabilities. Models were developed using various combinations of constraints including time, behaviour after first capture, and age. For age models, we considered that young snow leopards turned into adults after the age of 2 years. We tested if the parameters for these young snow leopards remained the same after 2 years, became similar to other known adults, or were entirely different from young and adult snow leopards. Models were compared using the corrected Akaike Information Criteria (AICc) and the final estimates were obtained using model averaging following recommendations by [29] on model selection. Variances of the model-averaged results were obtained using methods prescribed by [30].

## Results

Of 381 snow leopard encounters over the four primary sampling periods, we could identify the individuals in 361 (95%). In total we used data from 13 individuals marked as adults and 21 marked as young in our analysis across four years. The adult population was estimated separately for each year using photographic data of 8, 11, 13 and 13 adult snow leopards identified respectively for each year.

For the multi-season models, heterogeneity in detection of individuals did not rank high among the most parsimonious models. However the models indicated that detection probability for secondary sampling occasions of individuals marked as adults (0.16+−SE 0.02) was greater than that of young individuals (0.11+−SE 0.02). AIC model weights indicated little difference between capture and recapture probabilities, suggesting no behavioural effect of camera trapping.

Populations closure test for all the four years were consistent with the assumption that the snow leopard population was closed during the primary sampling periods (Table 1). Jackknife estimation of abundance using the heterogeneity models [25] showed that the estimated mean adult population ranged from 12 (95% CI = 11–19) to 14 (95% CI = 14–21) during the four years (Table 1). AIC weights from the robust design analysis models (Table 2) indicated there was greater than 95% probability that the adult population remained constant over four years. Pradel's population growth rate model also suggested that the population had remained stable across the four years (λ = 1.08+−0.25). Our mark-recapture estimate for the population subset comprising of radio-collared individuals was identical to the actual ‘known population’ in each of the four years (7, 10, 8 and 10 individuals, respectively), indicating that our overall population estimates may have been accurate (Table 3).

**Table 1:** Abundance of snow leopards estimated using the Jackknife estimator on camera trapping data in Tost Mountains, South Gobi, Mongolia, between 2009 and 2012.

Year			Adult			Adult+Young		
				95% Confidence Interval			95% Confidence Interval	
	z	p_z_	N	LCL	UCL	N	LCL	UCL
2009	−0.38	0.35	12	10	21	20	17	35
2010	−1.41	0.13	14	13	17	20	19	26
2011	−0.42	0.34	12	11	15	19	17	23
2012	0.36	0.64	14	14	21	21	21	31

Abundance of Adult+Young snow leopards was estimated by multiplying the estimates of Adult abundance by mean group size of each identified snow leopard. z = Population closure test statistic, p_z_ = closure test significance, N = Abundance, LCL = lower confidence limit, and UCL = upper confidence limit.

**Table 2:** Top 10 models (cumulative AIC weight, CW = 0.98) in the robust design analysis of multi-season camera trapping of snow leopards for variables affecting survival (S), abundance (N), temporary immigration (G1), temporary emigration (G2), capture probability (p), and recapture probability (c) for snow leopards in the Tost Mountains, South Gobi, Mongolia, between 2009 and 2012.

Model	AICc	Δ AICc	W	Model Likelihood	K	Model Deviance	CW
{S(.) G1 = G2 (AgeGrp) p = c(AgeGrp) N(.)}	659.4	0	0.237	1	6	638.289	0.24
{S(.) G1 = G2 (Yng-Ad_2yr&diff) p = c(AgeGrp) N(.)}	659.44	0.0239	0.234	0.9881	7	636.084	0.47
{S(.) G1 = G2 (AgeGrp) p = c(AgeGrp) N(Age)}	660.12	0.6998	0.167	0.7048	7	636.760	0.64
{S(Yng-Ad_2yr&diff) G1 = G2 (Yng-Ad_2yr&diff) p = c(AgeGrp) N(.)}	661.50	2.0813	0.084	0.3532	8	635.876	0.72
{S(Yng→Ad_2yr) G1 = G2 (AgeGrp) p = c(AgeGrp) N(.)}	661.60	2.1781	0.080	0.3365	7	638.238	0.80
{S(Yng→Ad_2yr) G1 = G2 (AgeGrp) p = c(AgeGrp) N(Age)}	662.33	2.9139	0.055	0.233	8	636.709	0.86
{S(.) G1 = G2 (Yng→Ad_2yr) p = c(AgeGrp) N(.)}	662.63	3.2157	0.047	0.2003	6	641.505	0.90
{S(.) G1 = G2 (Yng→Ad_2yr) p = c(AgeGrp) N(Age)}	663.04	3.6172	0.039	0.1639	7	639.677	0.94
{S(.) G1 = G2 (AgeGrp) p = c(AgeGrp) N(Session)}	663.57	4.1541	0.030	0.1253	9	635.647	0.97
{S(Yng→Ad_2yr) G1 = G2 (AgeGrp) p = c(AgeGrp) N(Session)}	665.86	6.4427	0.009	0.0399	10	635.596	0.98

Included in the table are: AICc = corrected Akaike Information Criteria, ΔAICc = differences in AICc values between each model and the best fitting model, *W* = model weight, Model Likelihood, K = number of model parameters, and Model Deviance. Constraints tested included Yng→Ad_2yr = snow leopards marked as young turn into adults after 2 years with parameters similar to the latter, Yng-Ad_2yr&diff = snow leopards marked as young turn into sub-adults after 2 years with parameters different from young as well as adults; AgeGrp = snow leopards marked as young have parameters different than for those marked as adult, Session or Period = conditions when parameters are considered to be different for each year.

**Table 3:** A comparison of the known ‘population’ of radio-collared snow leopards and their population estimated using mark-recapture analysis in the Tost Mountains, South Gobi, Mongolia, between 2009 and 2012.

Year	Known population of collared individuals	Camera trapped collared individuals	Mark-Recapture estimates of collared population	Std. Err.	95% LCL	95% UCL
2009	7	5	7.33	2.32	5.45	16.85
2010	10	9	9.83	1.43	9.08	17.3
2011	8	7	7.52	1.24	7.04	14.75
2012	10	10	10	0.29	10	10

Estimation of male and female ratios in the four primary sampling periods showed a significant change in adult sex ratio (χ^2^ = 5.8; p = 0.02) from 1.67 males per female in 2009 to 0.38 males per female in 2012 (Fig. 2). The proportion of females in the adult population increased from 0.38 (estimated 4 individuals) to 0.67 (10), whereas that of males declined from 0.63 (6) to 0.33 (4). The total number of cubs recorded on camera traps each year remained between 8 and 9, while the number of cubs per female declined from 2 in 2009 to 0.88 in 2012 as the female population increased (Fig. 2).

We found no evidence in support of models assuming variation in survival probability, indicating that snow leopard survival probability did not vary over the years (Table 2). This enabled us to use the reduced parameters approach and estimate survival probability of snow leopards for the entire study period. The model averaged estimate of survival probability of adults was 0.83 (SE+−0.08), and that of the individuals marked when young were 0.83 (SE+−0.15) and 0.77 (SE +−0.2) respectively, before and after the age of 2 years (Table 4). This meant that approximately 17% of the adult and young and 23% of the sub-adult snow leopards either died or permanently emigrated out of the study area annually. While we could estimate temporary emigration and immigration, our study design did not allow us to differentiate between permanent migration and mortality. However, assuming individuals that migrated permanently were lost from the study population (died), we estimated the life expectancy of adult snow leopards to be 5 years (95%CI: 2.05–13.78 years).

**Table 4:** Probabilities of annual survival and temporary emigration and immigration of adult and young snow leopards in the Tost Mountains, South Gobi, Mongolia, between 2009 and 2012.

	Survival (S)	SE	LCL	UCL	Emigration/Immigration (γ)	SE	LCL	UCL
Adult	**0.83**	0.075	0.614	0.926	**0.01**	0.07	0.00	0.15
Young (<2 yrs)	**0.83**	0.154	0.328	0.960	**0.60**	0.19	0.24	0.87
Young (>2 yrs)	**0.77**	0.201	0.264	0.968	**0.68**	0.32	0.11	0.97

SE = Standard Error, LCL = 95% Lower Confidence Limit, UCL = 95% Upper Confidence Limit.

For the entire dataset, the probability of temporary emigration and immigration appeared to be random rather than Markovian, indicating that it was not dependent on the previous state of the individuals. For individuals marked as young, the probability of random temporary emigration and immigration was estimated to be 0.6 (SE +−0.19) and 0.68 (SE +−0.32) respectively, before and after the age of 2 years, suggesting that an estimated 60–68% of young individuals emigrated from the study area, potentially returning subsequently (Table 4). However, there was little evidence of temporary emigration and immigration in the adult population (0.01 SE+−0.07).

## Discussion

In this first ever multi-year monitoring of snow leopards, we found the population in Tost Mountains of South Gobi to have remained almost constant, and the estimated mean adult population remained between 12 and 14, and the total population (including young) between 19 and 21. That our overall abundance estimates were reliable was supported by the accurate estimation of the known population of radio-collared snow leopards during the study period. The population also appeared fecund as we recorded confirmed births of at least 21 cubs in the study area over the 4 years, and estimated a total of 32 cubs after incorporating detection probability. Our estimate of annual survival of adult snow leopards (0.82±0.08) was comparable to annual adult survival probabilities reported for tigers in South India (0.77, [2]) and leopards in Kruger National Park, South Africa (0.82, [31]), both characterizing stable populations. Other studies with higher level of human induced mortality have reported relatively lower survival probabilities, such as in the case of male Amur tigers in southeast Russia (0.72, [32]), male cougars in the Pacific north-west America (0.55, [33]), and male leopards in South Africa (0.45, [34]), and predicted negative population growths. From these perspectives, the snow leopard population in Tost thus appeared to be stable and not in decline.

Yet, while the abundance estimates indicate a stable snow leopard population in Tost, a closer examination reveals vigorous underlying dynamics. For instance, the significant change in adult sex ratio of snow leopards over the sampling period we recorded was unexpected. We started with a male-biased population, and over the 4 years, it became female-biased with the proportion of males becoming almost half and that of the females in the population almost doubling. Indeed, in our radio-collaring efforts on the same population during the same study period, we physically captured only 1 adult female and 8 male snow leopards during the first two years, whereas in the subsequent years, 8 females and 6 males (including some that had been captured earlier) were captured [35], which may support our finding of the change in sex ratio. Disproportionate mortality of males, presumably an important cause of the change in sex ratio of the snow leopard population in Tost, is reported in several carnivores as resulting from human-induced factors such as poaching and retaliatory killing, including Amur tigers in southeast Russia [32], leopards in South Africa [34], and cougars in the Pacific Northwest [33], although there are exceptions (e.g. tiger population in Panna, Central India, that had turned male-biased prior to extinction due to poaching; [36], [37]). Males of large carnivores are known for their tendency to disperse further and range over much larger areas as compared to females who may remain in close vicinity to their mother's range after dispersal e.g. see [38], [39], exposing the males to greater anthropogenic risks. However in a fragmented habitat where dispersal distance could be limited by habitat restrictions hindering connections with a neighbouring population, the difference might not be so striking. During our 4 year study period, we recorded the mortality of 4 adult snow leopards, all of which were males. We could ascertain the cause of mortality only in one case, where the individual had been shot by a herder. This may be indicative of anthropogenic pressure on the Tost population.

The change in sex ratio of snow leopards in Tost was accompanied by a potential decline in the number of cubs per female over the study period as the female population increased. It is likely that young females dispersing around their natal ranges may have resulted in the proportion of females in the study area to increase while males suffered disproportionate mortality or dispersed to areas beyond the study area. The younger females perhaps do not reproduce in the first year or so after dispersal, possibly explaining the reduced number of cubs per female. With more data in the future on age at first reproduction, sexual differences in dispersal strategies, and connectivity between neighbouring habitats, we hope to be able to better understand the mechanisms behind these population dynamics in Tost.

The much lower random probability of temporary emigration and immigration we recorded for adult snow leopards points to their greater site fidelity and perhaps territoriality. Expectedly, as compared to the adults, we recorded a relatively higher rate of temporary emigration and immigration by young snow leopards, presumably due to dispersal attempts and efforts to establish their territories outside their natal areas as observed in other cat species [40], [41]. Although the Tost Mountains appear relatively isolated, it appears that young individuals use the surrounding landscape for dispersal, despite the absence of contiguous, large mountain chains. Two young collared snow leopards illustrated this by dispersing to the nearby Nemegt Mountains after crossing about 80 km of steppe. The movements between landscapes through steppe underscore the need for paying attention to the intervening steppe habitats of Central Asia while planning for snow leopard conservation.

From a methodological perspective, we achieved high (95%) success in identification of individual snow leopards from photographs, even though we used only one camera per trapping station. As long as trapping stations are set up in sites where snow leopards are likely to linger (e.g. marking locations), thereby allowing multiple images and both flanks to be photographed, one camera trap appears adequate, especially when conducting long-term monitoring and using cameras that are capable of taking multiple images rapidly (<1 second intervals). This enables twice the area to be sampled with a given number of cameras. Indeed many of our snow leopard encounters that remained unidentified during the initial years could be identified in due course over multi-year sampling. For studies of a shorter duration, or one-time sampling of the population, one camera might not always be adequate. Although all snow leopards were identified as unique individuals based on camera trap pictures, the sexing of adults would perhaps not have been possible for many individuals (except females with young), had we not been capturing them for collaring.

Our work, apart from providing the first estimates of vital rates and other population characteristics of the endangered snow leopard, reiterates the value of long-term monitoring of both abundance and population dynamics for conservation planning and action.

## References

[1] Williams BK, Nichols JD, Conroy MJ (2002) Analysis and management of animal populations. 1st ed. San Diego, California: Academic Press.

[2] Karanth KU, Nichols JD, Kumar NS, Hines JE (2006) Assessing tiger population dynamics using photographic capture-recapture sampling. Ecology 87: 2925–2937 Available: http://www.ncbi.nlm.nih.gov/pubmed/17168036 17168036 10.1890/0012-9658(2006)87[2925:atpdup]2.0.co;2

[3] Kerley LL, Goodrich JM, Miquelle DG, Smirnov EN, Quigley HB, et al (2003) Reproductive parameters of wild female Amur (Siberian) tigers (Panthera tigris altaica). J Mammal 84: 288–298 Available: http://www.asmjournals.org/doi/abs/10.1644/1545-1542(2003)084<0288:RPOWFA>2.0.CO;2. Accessed 8 November 2013

[4] Caro TM (2003) Umbrella species: critique and lessons from East Africa. Anim Conserv 6: 171–181 Available: http://doi.wiley.com/10.1017/S1367943003003214. Accessed 30 July 2012

[5] Treves A, Karanth KU (2003) Human-Carnivore Conflict and Perspectives on Carnivore Management Worldwide. Conserv Biol 17: 1491–1499 Available: http://doi.wiley.com/10.1111/j.1523-1739.2003.00059.x.

[6] Karanth KU, Chellam R (2009) Carnivore conservation at the crossroads. Oryx 43: 1 Available: http://www.journals.cambridge.org/abstract_S003060530843106X. Accessed 8 November 2013

[7] Gittleman JL, Harvey PH (1982) Carnivore home-range size, metabolic needs and ecology. Behav Ecol Sociobiol 10: 57–63 doi:10.1007/BF00296396

[8] Karanth K (1995) Estimating tiger Panthera tigris populations from camera-trap data using capture—recapture models. Biol Conserv 71: 333–338 Available: http://www.sciencedirect.com/science/article/pii/000632079400057W. Accessed 30 January 2014

[9] Mills LS, Citta JJ, Lair KP, Schwartz MK, Tallmon DA (2000) Estimating animal abundance using noninvasive DNA sampling: promise and pitfalls. Ecol Appl 10: 283–294 Available: http://www.esajournals.org/doi/abs/10.1890/1051-0761(2000)010[0283:EAAUND]2.0.CO;2. Accessed 8 November 2013

[10] Wallace RB, Gomez H, Ayala G, Espinoza F (2003) Camera trapping for jaguar (Panthera onca) in the Tuichi Valley, Bolivia. J Neotrop Mammal 10: 133–139 Available: http://www.sarem.org.ar/wp-content/uploads/2012/11/SAREM_MastNeotrop_10-1_10_Wallace.pdf. Accessed 12 January 2014

[11] Silver S, Ph D (2004) Assessing jaguar abundance using remotely triggered cameras. Wildl Conserv

[12] Maffei L, Cuéllar E, Noss A (2004) One thousand jaguars (Panthera onca) in Bolivia's Chaco? Camera trapping in the Kaa-Iya National Park. J Zool 262: 295–304 Available: http://onlinelibrary.wiley.com/doi/10.1017/S0952836903004655/abstract. Accessed 8 November 2013

[13] Jackson RM, Roe JO, Wangchuk R, Hunter DO (2006) Estimating Snow Leopard Population Abundance Using Photography and Capture – Recapture Techniques. Wildl Soc Bull 34: 772–781.

[14] Athreya V, Odden M, Linnell JDC, Krishnaswamy J, Karanth U (2013) Big Cats in Our Backyards: Persistence of Large Carnivores in a Human Dominated Landscape in India. PLoS One 8: e57872 Available: http://dx.plos.org/10.1371/journal.pone.0057872. Accessed 7 March 2013 23483933 10.1371/journal.pone.0057872 PMC3590292

[15] Marnewick K, Funston PJ, Karanth KU (2008) Evaluating camera trapping as a method for estimating cheetah abundance in ranching areas. South African J Wildl Res 38: 59–65 Available: http://www.bioone.org/doi/abs/10.3957/0379-4369-38.1.59

[16] Trolle M, Kéry M (2003) Estimation of ocelot density in the Pantanal using capture-recapture analysis of camera-trapping data. J Mammal 84: 607–614 Available: http://www.asmjournals.org/doi/abs/10.1644/1545-1542(2003)084<0607:EOODIT>2.0.CO;2. Accessed 8 November 2013

[17] Maffei L, Noss AJ, Ceullar E, Rumiz DI (2005) Ocelot (Felis pardalis) population densities, activity, and ranging behaviour in the dry forests of eastern Bolivia: data from camera trapping. J Trop Ecol 21: 1–6.

[18] Mccarthy TM, Chapron G (2003) Snow Leopard Survival Strategy. Seattle, USA: ISLT and SLN.

[19] Mccarthy KP, Fuller TK, Ming M, M MT, Waits L, et al (2008) Assessing Estimators of Snow Leopard Abundance. J Wildl Manage 72: 1826–1833 Available: http://onlinelibrary.wiley.com/doi/10.2193/2008-040/abstract. Accessed 8 November 2013

[20] Janečka JE, Munkhtsog B, Jackson RM, Naranbaatar G, Mallon DP, et al (2011) Comparison of noninvasive genetic and camera-trapping techniques for surveying snow leopards. J Mammal 92: 771–783 Available: http://www.bioone.org/doi/abs/10.1644/10-MAMM-A-036.1. Accessed 2 March 2013

[21] Shehzad W, Mccarthy TM, Pompanon F, Purevjav L, Coissac E, et al (2012) Prey preference of snow leopard (Panthera uncia) in South Gobi, Mongolia. PLoS One 7: 1–8 Available: http://dx.plos.org/10.1371/journal.pone.0032104. Accessed 12 January 2014 10.1371/journal.pone.0032104 PMC3290533 22393381

[22] Karanth K, Nichols J (1998) Estimation of tiger densities in India using photographic captures and recaptures. Ecology 79: 2852–2862 Available: http://www.esajournals.org/doi/abs/10.1890/0012-9658(1998)079 [2852:EOTDII]2.0.CO;2. Accessed 12 January 2014

[23] Nichols JD, Karanth KU (2002) Statistical concepts: estimating absolute densities of tigers using capture–recapture sampling. In: Nichols JD, Karanth KU, editors. Monitoring tigers and their prey: amanual for researchers,managers and conservationists in tropical Asia. Bangalore: Centre for Wildlife Studies. pp. 121–137.

[24] Karanth KU, Chundawat RS, Nichols JD, Kumar NS (2004) Estimation of tiger densities in the tropical dry forests of Panna, Central India, using photographic capture–recapture sampling. Anim Conserv 7: 285–290 Available: http://onlinelibrary.wiley.com/doi/10.1017/S1367943004001477/abstract. Accessed 8 November 2013

[25] Otis DL, Burnham KP, White GC, Anderson DR (1978) Statistical inference from capture data on closed animal populations. Wildl Monogr 62: 3–135.

[26] Pradel R (1996) Utilization of capture-mark-recapture for the study of recruitment and population growth rates. Biometrics 52: 703–709.

[27] Kendall WL, Pollock KH, Brownie C (1995) A Likelihood-Based Approach to Capture-Recapture Estimation of Demographic Parameters under the Robust Design A Likelihood-Based Approach to Capture-Recapture Estimation of Demographic Parameters Under the Robust Design. Biometrics 51: 293–308. 7766783

[28] Kendall WL, Bjorkland R (2001) Using Open Robust Design Models to Estimate Temporary Emigration from Capture-Recapture Data Using Open Robust Design Models to Estimate Temporary Emigration from Capture-Recapture Data. Biometrics 57: 1113–1122. 11764251 10.1111/j.0006-341x.2001.01113.x

[29] Burnham KP, Anderson DR, Huyvaert KP (2010) AIC model selection and multimodel inference in behavioral ecology: some background, observations, and comparisons. Behav Ecol Sociobiol 65: 23–35 Available: http://link.springer.com/10.1007/s00265-010-1029-6. Accessed 22 May 2013

[30] Cooch E, White G (2008) Program Mark: a gentle introduction. 7th ed. Cooch E, White G, editors.

[31] Bailey TN (1993) The African leopard: Ecology and Behaviour of a solitary felid. New York: Columbia University Press.

[32] Goodrich JM, Kerley LL, Smirnov EN, Miquelle DG, McDonald L, et al (2008) Survival rates and causes of mortality of Amur tigers on and near the Sikhote-Alin Biosphere Zapovednik. J Zool 276: 323–329 Available: http://doi.wiley.com/10.1111/j.1469-7998.2008.00458.x. Accessed 13 August 2013

[33] Lambert CMS, Wielgus RB, Robinson HS, Katnik DD, Cruickshank HS, et al (2006) Cougar population dynamics and viability in the Pacific Northwest. J Wildl Manage 70: 246–254 Available: http://www.bioone.org/doi/abs/10.2193/0022-541X(2006)70[246:CPDAVI]2.0.CO;2. Accessed 8 November 2013

[34] Balme G, Hunter L (2004) Mortality in a protected leopard population, Phinda Private Game Reserve, South Africa: A population decline. Ecol J 6: 1–6.

[35] Johansson Ö, Malmsten J, Mishra C, Lkhagvajav P, McCarthy T (2013) Reversible immobilization of free-ranging snow leopards (panthera uncia) with a combination of medetomidine and tiletamine-zolazepam. J Wildl Dis 49: 338–346 Available: http://www.ncbi.nlm.nih.gov/pubmed/23568909. Accessed 7 November 2013 23568909 10.7589/2012-02-049

[36] Chundawat RS, Gruisen J (2009) Panna's last tiger. New Delhi: BAAVAN-Baagh Aap Aur Van.

[37] Gopal R, Qureshi Q, Bhardwaj M, Jagadish Singh RK, Jhala YV (2010) Evaluating the status of the endangered tiger Panthera tigris and its prey in Panna Tiger Reserve, Madhya Pradesh, India. Oryx 44: 383–389.

[38] Smith JLD (1993) The Role of Dispersal in Structuring the Chitwan Tiger Population. Behaviour 124: 165–195 doi:10.1163/156853993X00560

[39] Gour DS, Bhagavatula J, Bhavanishankar M, Reddy PA, Gupta Ja, et al (2013) Philopatry and dispersal patterns in tiger (Panthera tigris). PLoS One 8: e66956 Available: http://www.pubmedcentral.nih.gov/articlerender.fcgi?artid=3699573&tool=pmcentrez&rendertype=abstract 23843973 10.1371/journal.pone.0066956 PMC3699573

[40] Samelius G, Andren H, Liberg O, Linnel JDC, Odden J, et al (2012) Spatial and temporal variation in natal dispersal by Eurasian lynx in Scandinavia. J Zool 286: 120–130.

[41] Vangen KM, Persson J, Landa A, Anderson R, Segerstrom P (2001) Characteristics of dispersal in wolverines. Can J Zool 79: 1641–1649.

